# Single‐cell mRNA sequencing of giant panda (*Ailuropoda melanoleuca*) seminoma reveals the cellular and molecular characteristics of tumour cells

**DOI:** 10.1002/vms3.1348

**Published:** 2024-01-16

**Authors:** Chen Yijiao, An Junhui, Hou Rong, Liu Yuliang, Wang Donghui, Liu Songrui, Feng Tongying

**Affiliations:** ^1^ Chengdu Research Base of Giant Panda Breeding Chengdu China; ^2^ Sichuan Key Laboratory of Conservation Biology for Endangered Wildlife Chengdu China; ^3^ Sichuan Academy of Giant Panda Chengdu Chengdu China

**Keywords:** giant panda, reproduction, seminoma, single‐cell mRNA sequencing, tumour

## Abstract

Testicular tumours are zoonoses that can occur in not only human, but other animals, include giant pandas. A middle‐aged male giant panda named Fufu was diagnosed with a testicular tumour and underwent surgery to remove the entire left testis. The testis was mainly composed of three substantive parts: normal tissue on the outside, tumour tissue in the middle, and necrosis in the centre. HE stains revealed that the tumour was a seminoma.

Single‐cell mRNA sequence was applied to characterise cellular states and molecular circuitries of giant panda testicular seminoma. Only germ cell markers expressed in nearly all tumour cells, and the tumour cells appeared to be the same subtype of seminoma cells.

We identified four clusters with unique genes expression. They were early apoptosis cells (EAC), inactive cells (IC), active cells subcluster 1 (AC‐1) and active cells subcluster 2 (AC‐2). We utilised monocle tools and found that IC cells was in the initiation stage, and EAC was one type of terminal stage, suggesting that tumour cells may undergo apoptosis in the future. AC‐2 was another type of terminal stage, representing a group of progressive cells.

Our study represents the first report to utilise scRNA‐seq to characterise the cellular states and molecular circuitries of a giant panda testicular tumour. This investigation proposes CD117 and CD30 as dependable markers for future pathologic diagnosis. Our findings also suggest that CTSV and other genes with unique expression patterns in active and progressive giant panda seminoma cells may act as early prognostic biomarkers.

## Graphical abstract

Four subclusters of giant panda seminoma tumour cells with unique gene expression were found.

Two distinct tumour developmental pathways were found: one is apoptosis and the other one is progress of progressive tumour. Four pivotal genes significantly influencing the developmental trajectory: CDKN1A, CENPF, DLGAP5 and MKI67.

CD117 and CD30 as dependable markers for future giant panda seminoma pathologic diagnosis.

CTSV, TPT1, MAEL, CITED2 and TKTL1 may be used as potential early prognostic biomarkers for giant panda cancer/tumour.

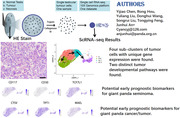

1

In human, testicular tumours can be divided roughly into germ cell tumours (GCT), sex cord‐stromal tumours (SCST), mixed tumours and secondary testicular tumours (which are extremely rare). SCST include　Leydig tumours, Sertoli tumours, Granulosa tumours and mixed types. GCT are classified in two main subtypes, seminoma and non‐seminoma due to histologic and clinical prognosis and therapeutic criteria, the latter of which contains embryonal teratoma, carcinoma, choriocarcinoma and yolk sac tumours (Al‐Obaidy & Idrees, 2021). Seminoma is the most common subtype of testicular GCT, which can either be pure or present in combination with other histotypes (Elzinga‐Tinke et al., 2015).

Testicular tumours are zoonoses that can occur in various species such as dogs, cats, bears, bulls, stallions, rabbits, roosters and giant pandas. Giant pandas Xing Xing (National Zoo in Washington, 1997), Gao Gao (San Diego Zoo, 2014) and Yang Guang were all diagnosed with testicular tumours and received testicular removal surgeries in the past decades. However, no further exploration of giant panda testicular tumours has been done, and thus the tumour types, main pathogenesis, gene expression characteristics, and early detection methods remain largely unknown.

In 2018, a middle‐aged male giant panda was diagnosed with a testicular tumour. His left testis was twice the size of the normal testis and had become harder. In February 2019, he underwent surgery to remove the entire left testis, which was mainly composed of three substantive parts: normal tissue on the outside, tumour tissue in the middle and necrosis in the centre (Figure 1A). Normal testicular tissue exhibited clear and intact seminiferous tubules (Figure 1B), while tumour tissue did not (Figure 1C). The tumour consisted of medium‐sized tumour cells with round or polygonal shapes, and their borders were not very clear. Their cytoplasm was slightly eosinophilic, with an eccentric, slightly eosinophilic nucleus with one or more prominent nucleoli, presenting typical seminoma cell morphology. To further understand the biological characteristics and dynamics within tumour/cancer lesions, single‐cell mRNA sequence (scRNA‐seq) was applied in this study, which was a powerful tool to effectively delineate cell types, uncover heterogeneity and infer developmental trajectories.

**FIGURE 1:**
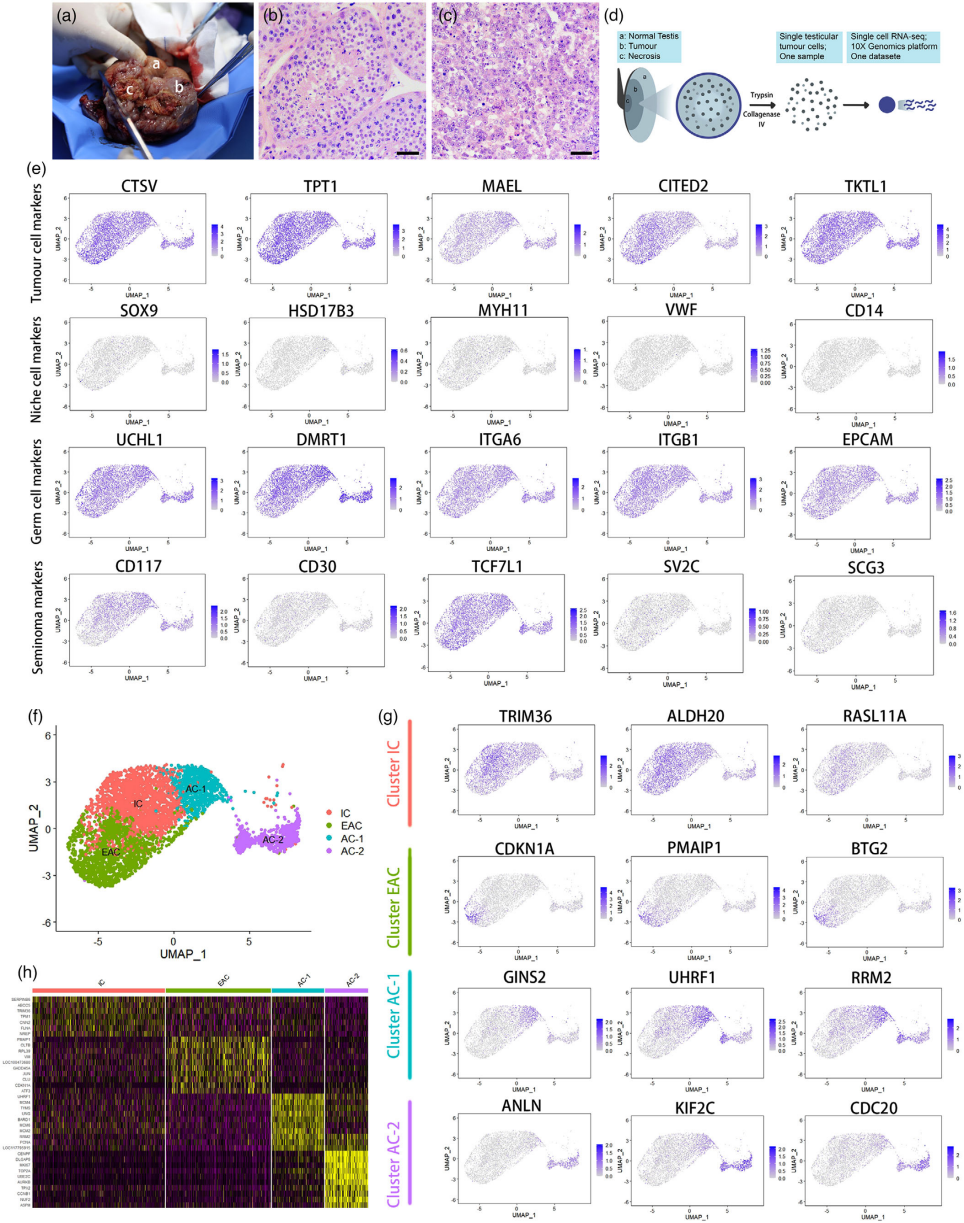
**(A)** Left testis of Fufu, a was normal testis tissue, b was tumour, and c was necrosis. **(B)** Photomicrographs of HE stained Fufu's normal testis (section a in A), with intact seminiferous tubules. Scale bar, 100 μm. **(C)** Photomicrographs of HE stained Fufu's tumoural section (section b in A), cells were large, exhibited round or polygonal shape, with hyperchromatic cytoplasm and increased nuclear chromatin. Scale bar, 100 μm. **(D)** Schematic illustration of the experimental workflow. **(E)** UMAP plot exhibition per‐cell expression of known or novel markers of the giant panda specific cell types. **(F)** UMAP plot illustrating 4674 seminoma cells that were allocated into 4 clusters. **(G)** UMAP plot showing per‐cell expression patterns genes. **(H)** The heatmap of the seminoma cells in each cell cluster. Colours from yellow to magenta represent the expression level from high to low.

In this study, cells from the centre of the tumour were digested using 0.25% Trypsin and 1 mg/mL Collagenase Type IV. Subsequently, they were loaded onto the 10× Genomics platform to perform scRNA‐seq (Figure 1D). The raw data were obtained directly from the 10× Genomics platform. Cell Ranger, which is a software package provided by 10× Genomics, was utilised for analysing. Cell Ranger compared the generated raw data to the reference genome for UMI counting and performs barcode filtering based on the distribution of UMI to generate the gene expression matrix of each cell. After applying standard quality control (QC) dataset filters, approximately 4674 cells were retained for downstream analysis. The mean reads/cell of 102,479 allowed for the analysis of a median of 5452 genes/cell. To determine cell types, specific markers with consistent expression in nearly all cells were inspected based on previous reports (by R package Seurat v1.4.3.0). As shown in Figure 1E, the high expression of markers (Abbreviation of genes and their official full names were listed in Table 1) such as CTSV, TPT1, MAEL, CITED2 and TKTL1 reliably assigned these cells as tumour cells. Interestingly, analysis of specific somatic cell markers indicated that there were no somatic cells in the tumour tissue, as evidenced by the absence of SOX9 (Sertoli cell marker), HSD17B3 (Leydig cell marker), MYH11 (Myoid cell marker), VWF (Endothelial cell marker) and CD14 (Macrophage cell marker) expression. In contrast, germ cell markers UCHL1, DMRT1, ITGA6, ITGB1 and EPCAM were expressed in nearly all tumour cells. According to previous reports, CD117 and CD30 are reliable markers used in combination for testis germ cell tumour diagnosis. We found that Fufu's testicular tumour had high expression of CD117 but low expression of CD30 (Figure 1E), which was in consistent with previous reports that most seminomas were CD117+/CD30‐ (Leroy et al., 2002). The research indicates that TCF7L and SVC2/SCG3 can be used to distinguish subtypes of seminoma (Mo et al., 2022). The fact that Fufu's seminoma expresses TCF7L but does not express SVC2/SCG3 suggested that it belonged to a unique subtype characterised by TCF7L expression alone.

**TABLE 1:** Abbreviation of genes.

Official symbol	Official full name
CTSV	Cathepsin V
TPT1	Tumour protein, translationally controlled 1
MAEL	Maelstrom spermatogenic transposon silencer
CITED2	Cbp/p300‐interacting transactivator, with Glu/Asp‐rich carboxy‐terminal domain, 2
TKTL1	Transketolase like 1
SOX9	SRY‐box transcription factor 9
HSD17B3	Hydroxysteroid 17‐beta dehydrogenase 3
MYH11	Myosin heavy chain 11
VWF	Von Willebrand factor
CD14	CD14 molecule
UCHL1	Ubiquitin C‐terminal hydrolase L1
DMRT1	Doublesex and mab‐3‐related transcription factor 1
IGTA6	Integrin subunit alpha 6
ITGB1	Integrin subunit beta 1
EPCAM	Epithelial cell adhesion molecule
CD117	KIT proto‐oncogene, receptor tyrosine kinase
CD30	TNF receptor superfamily member 8
TCF7L1	Transcription factor 7 like 1
SV2C	Synaptic vesicle glycoprotein 2c
SCG3	Secretogranin III
TRIM36	Tripartite motif containing 36
ALDH2	Aldehyde dehydrogenase 2 family member
RASL11A	RAS like family 11 member A
CDKN1A	Cyclin‐dependent kinase inhibitor 1A
PMAIP1	Horbol‐12‐myristate‐13‐acetate‐induced protein 1
BTG2	BTG anti‐proliferation factor 2
GINS2	GINS complex subunit 2
UHRF1	Ubiquitin like with PHD and ring finger domains 1
RRM2	Ribonucleotide reductase regulatory subunit M2
ANLN	Anillin actin binding protein
KIF2C	Kinesin family member 2C
CDC20	Cell division cycle 20
CENPF	Centromere protein F
DLGAP5	DLG associated protein 5
MKI67	Marker of proliferation Ki‐67

Unsupervised clustering identified 4 clusters (Figure 1F), they were early apoptosis cells (EAC), inactive cells (IC), active cells subcluster 1 (AC‐1) and active cells subcluster 2 (AC‐2). Three typical genes of each cluster were selected and shown in UMAP plot (Figure 1G). CDKN1A, PMAIP1 and BTG2 were solely expressed EAC cluster, and the overexpression of them can result in cell cycle arrest in the G0/G1 phase, induce the apoptosis of cancer cells and inhibit cell growth. In IC cluster, the expression of TRIM36, ALDH2 and RASL11A was relatively high, and in vitro experiment had suggested that the overexpression of them can significantly inhibit tumour growth, proliferation, stemness and migration (Liang et al., 2018). By contrast, AC‐1 were in the progress of cell proliferation, characterised by high expression of GINS2 and UHRF1, which can accelerate the growth of tumour cells and promote aerobic glycolysis (Tian et al., 2020). What's more, active cells also overexpressed RRM2 to increase their drug resistance ability (Apostolidis et al., 2012). AC‐2 showed particularly high expression of ANLN, KIF2C and CDC20, suggesting that they may undergo malignant progression of tumour/cancer. ANLN could promote epithelial to mesenchymal transformation (EMT) while KIF2C could accelerate progression and impeded apoptosis (Liu et al., 2019).

When comparing clusters, we observed relatively distinct characteristics within the IC, EAC, AC‐1 and AC‐2 clusters (Figure 2A). Cell cycle analyse revealed that IC and EAC clusters predominantly resided in the G1 phase, whereas AC‐1 cluster was primarily positioned in the S phase, and nearly all cells in AC‐2 cluster were found in the G2 phase (as illustrated in Figure 2B). The top 100 marker genes of each cluster were selected to do enrichment analysis. We also identified significant enrichments in various biological processes across all four clusters, with a notable emphasis on cell cycle‐related processes, including ‘Mitotic cell cycle’, ‘DNA replication’, ‘Chromosome organisation’ and ‘Chromosome condensation’ (as depicted in Figure 2C). Notably, the EAC cluster exhibited a unique enrichment in ‘Regulation of apoptotic signalling pathway’, while AC‐2 cluster exclusively displayed ‘Cell division’. Our analysis did not reveal any genes enriched in GO terms related to ‘developmental process’ and ‘anatomical structure morphogenesis’, which were observed in human aggressive seminoma (Mo et al., 2022). We utilised R package Monocle2 to reconstruct their developmental trajectory. This analysis indicated that IC cells mark the initiation stage. As EAC cells progress towards a terminal stage, they undergo apoptosis, which may be a component of the body's response to tumour rejection (Hanahan & Weinberg, 2011). Meanwhile, AC‐2 cells, also in a terminal stage, exhibit heightened cell proliferation, signifying the progression of tumour cells into an active state (refer to Figure 2D). These two directions represent distinct tumour developmental pathways (Morana et al., 2022). Through trajectory analysis, we also identified four pivotal genes significantly influencing the developmental trajectory: CDKN1A, CENPF, DLGAP5 and MKI67 (refer to Figure 2E).

**FIGURE 2:**
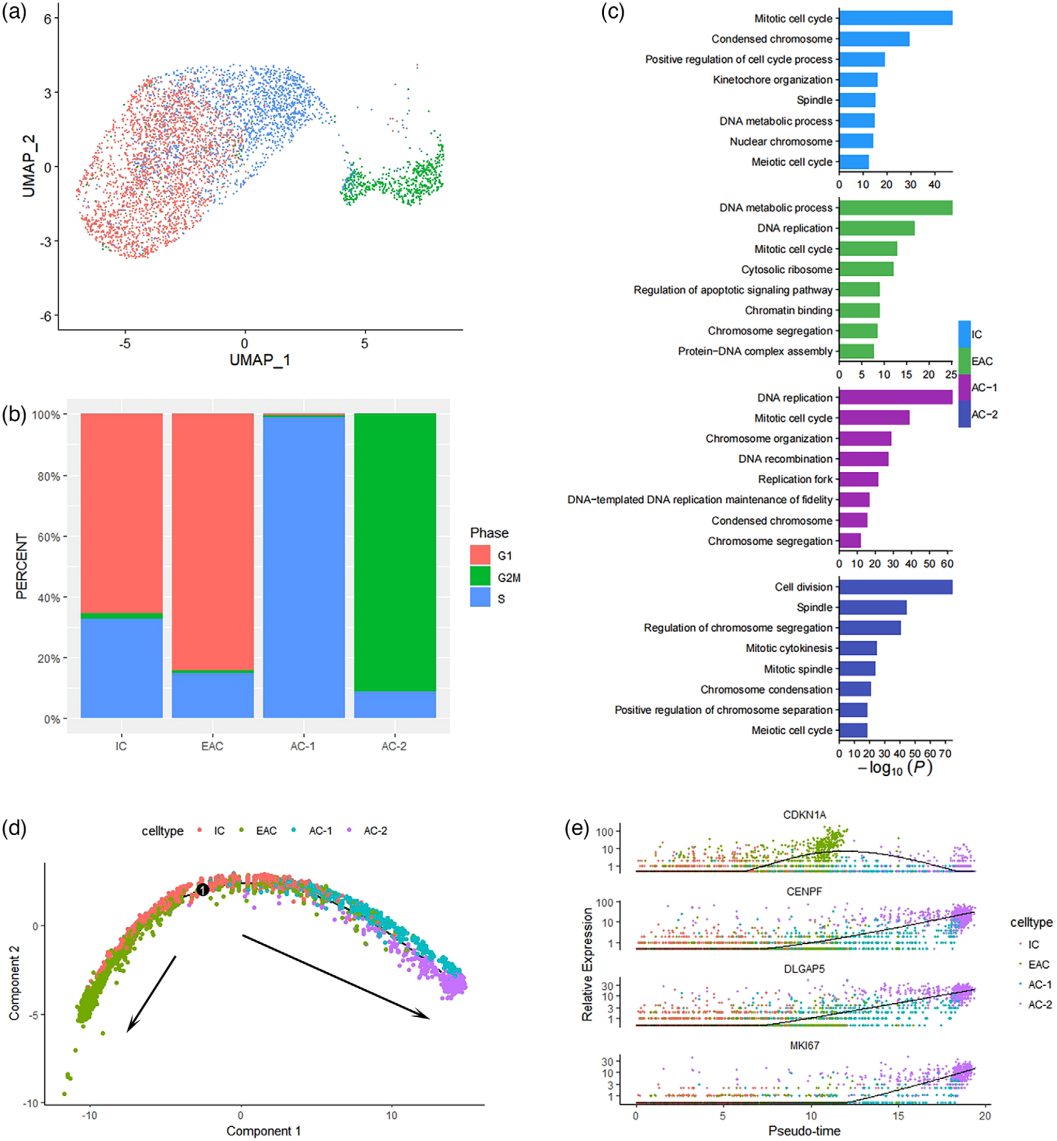
**(A)** UMAP plot illustrating the cell cycle phase of each seminoma cell. **(B)** The per cent of cell cycle phase in each cluster. **(C)** GO enrichment analysis of each cluster. **(D)** Reconstruction of the cell fate decisions and pseudotime trajectories of all seminoma cells by Monocle2. **(E)** Scatter plots showing the expression levels and changes in relative expression of key genes that affected the evolution stage.

Our study represents the first report to utilise scRNA‐seq to characterise the cellular states and molecular circuitries of a giant panda testicular tumour. This investigation confirms that Fufu was diagnosed with seminoma and proposes CD117 and CD30 as dependable markers for future pathologic diagnosis. Furthermore, a group of progressive markers was identified, suggesting that Fufu's testicular removal surgery was timely, preventing further malignancy, invasion or metastasis. Currently, Fufu is in good prognostic condition with no observable abnormalities in his body or behaviours, though as a high genetic value individual, the loss of his reproductive ability is a significant blow to the captive giant panda population. Despite seminoma being one of the most treatable cancers, with nearly 90% of patients having a good prognosis, lymph node metastasis often occurs, which can threaten their lives (Chung & Warde, 2011). Therefore, early detection and treatment are crucial. Thus, developing early prognostic biomarkers not only for giant panda seminoma but also for other kinds of cancers/tumours is urgent. Our findings suggest that CTSV and other genes (Figure 1E) with unique expression patterns in active and progressive giant panda seminoma cells may act as early prognostic biomarkers. Serum CTSV was found to distinguish lung cancer patients from healthy donors in previous studies (Yang et al., 2022), and other genes may have the potential to be developed as diagnostic markers for early pro‐invasive diagnosis in giant panda seminoma, which could benefit the welfare of giant pandas in the future.

## AUTHOR CONTRIBUTIONS

**Chen Yijiao**: data curation; writing – original draft; writing – review & editing. **An Junhui**: funding acquisition; supervision; writing – review & editing. **Hou Rong**: conceptualisation; supervision. **Liu Yuliang**: supervision. **Wang Donghui**: writing – review & editing. **Liu Songrui**: resources. **Feng Tongying**: software.

## CONFLICT OF INTEREST STATEMENT

All authors agree to declare that they have no conflicts of interest.

## FUNDING INFORMATION

Chengdu Giant Panda Breeding Research Foundation CPF2017‐15; Chengdu Research Base of Giant Panda Breeding 2020CPB‐B02; Sichuan Science and Technology Program 2020JDJQ0074.

## WRITTEN CONSENT STATEMENT

All procedures were conducted under the supervision of the Institutional Animal Care and Use of Chengdu Research Base of Giant Panda Breeding. The manuscript is approved by all authors for publication.

## ETHICS STATEMENT

“Single‐Cell mRNA Sequencing of Giant Panda (Ailuropoda melanoleuca) Seminoma Reveals the Cellular and Molecular Characteristics of Tumor Cells”, no conflict of interest exits in the submission of this manuscript, and manuscript is approved by all authors for publication. I would like to declare on behalf of my co‐authors that the work described was original research that has not been published previously, and not under consideration for publication elsewhere, in whole or in part.

### PEER REVIEW

The peer review history for this article is available at https://publons.com/publon/10.1002/vms3.1348.

## Data Availability

Data openly available in a public repository that issues datasets with DOIs.
